# A/H1N1 hemagglutinin antibodies show comparable affinity in vaccine-related Narcolepsy type 1 and control and are unlikely to contribute to pathogenesis

**DOI:** 10.1038/s41598-021-83543-z

**Published:** 2021-02-18

**Authors:** Alexander Lind, Ilaria Marzinotto, Cristina Brigatti, Anita Ramelius, Lorenzo Piemonti, Vito Lampasona

**Affiliations:** 1 Department of Clinical Sciences, Clinical Research Center (CRC), Skåne University Hospital SUS, Lund University, Malmö, Sweden; 2 San Raffaele Diabetes Research Institute, IRCCS Ospedale San Raffaele, Via Olgettina 60, 20132 Milan, Italy

**Keywords:** Immunology, Vaccines, Neurological disorders, Biomarkers, Immunoprecipitation

## Abstract

An increased incidence of narcolepsy type 1 (NT1) was observed in Scandinavia following the 2009–2010 influenza Pandemrix vaccination. The association between NT1 and HLA-DQB1*06:02:01 supported the view of the vaccine as an etiological agent. A/H1N1 hemagglutinin (HA) is the main antigenic determinant of the host neutralization antibody response. Using two different immunoassays, the Luciferase Immunoprecipitation System (LIPS) and Radiobinding Assay (RBA), we investigated HA antibody levels and affinity in an exploratory and in a confirmatory cohort of Swedish NT1 patients and healthy controls vaccinated with Pandemrix. HA antibodies were increased in NT1 patients compared to controls in the exploratory (LIPS p = 0.0295, RBA p = 0.0369) but not in the confirmatory cohort (LIPS p = 0.55, RBA p = 0.625). HA antibody affinity, assessed by competition with Pandemrix vaccine, was comparable between patients and controls (LIPS: 48 vs. 39 ng/ml, p = 0.81; RBA: 472 vs. 491 ng/ml, p = 0.65). The LIPS assay also detected higher HA antibody titres as associated with HLA-DQB1*06:02:01 (p = 0.02). Our study shows that following Pandemrix vaccination, HA antibodies levels and affinity were comparable NT1 patients and controls and suggests that HA antibodies are unlikely to play a role in NT1 pathogenesis.

## Introduction

Influenza viruses, through yearly seasonal outbursts and sporadically by global pandemic outbreaks, are a major burden of public health. Between October 2009 and early 2010, to implement protective measures against a novel influenza pandemic, Sweden offered to the entire population free influenza-vaccination with Pandemrix, a vaccine containing components from inactivated A/California/7/2009 (H1N1)v-like strain (X-179A) flu virus plus adjuvants. Following this Pandemrix vaccination, a 5–14 fold increase in incidence of narcolepsy type 1 (NT1) in children and adolescents was confirmed in Sweden and other European countries^1–9^. An increased incidence of NT1 following the 2009 pandemic, was also reported in China without a relation to vaccination^10–13^.

NT1 is a rare sleep disorder that results from the degeneration of brain hypothalamus-restricted hypocretin neurons^14^, with this specific cell loss causing unstable transitions between wakefulness and sleep stages. The causes and triggering mechanism behind this specific hypocretin cell-death are not well defined^15,16^. With a typical young age at onset and an almost complete association with the HLA-DQB1*06:02:01 allele, implying a connection to antigen-presentation^17,18^, NT1 is assumed to be the likely consequence of an autoimmune process.

The Pandemrix vaccination, as an unexpected and time-restricted trigger could provide new knowledge into the processes underlying the disease. Previous European studies measuring antibodies against non-structural protein 1, a protein present in the wild-type pandemic virus that was not included in the Pandemrix vaccine, did not observe an association between humoral immunity to the 2009 influenza pandemic virus and NT1^19,20^. Prior studies instead linked the vaccine-induced NT1 to the antibody response against influenza nucleoprotein (NP), that is present in structurally altered forms in the Pandemrix formulation^21^, and reported a potential molecular mimicry with the hypocretin receptor 2^22^, as well as a lower affinity of antibodies from NT1 patients’ sera recognizing the NP-PR1934 (the influenza nucleoprotein variant present in the Pandemrix vaccine)^20^.

However, it is acknowledged that hemagglutinin (HA) is the major antigenic determinant during an influenza infection. Suggestions of T cell mediated molecular mimicry between HA and hypocretin have been investigated with conflicting conclusions^23–29^. In this study we analysed the antibody response to HA with respect to Pandemrix vaccination in NT1 patients and healthy vaccinated Swedish residents in an exploratory cohort of samples collected in 2012, and a second replicatory cohort of samples from additional NT1 patients and their family members collected in 2015–2017.

We measured HA antibodies using the Luciferase Immunoprecipitation System (LIPS) and Radiobinding Assay (RBA), two alternative immunoassay formats widely adopted for the study of autoantibodies, i.e. assays that are more likely to detect antibodies to conformational epitopes^30–32^. We previously showed that an RBA test, using ^35^S-radiolabled HA expressed in vitro by coupled transcription, detected increased HA antibodies levels among NT1 patients in the exploratory cohort as well as an association of HA antibodies with HLA-DQB1*06:02^18^. In light of the different antigen expression system adopted by LIPS, which yields a trimeric post-translationally modified HA, we used the two assays to explore: first, if the HA antibody association to NT1 was found only in samples collected close to the time of vaccination with Pandemrix, or persisted also in confirmatory cohort of patients collected at a later time-point; second, if the antibody affinity of the HA response might differ between NT1 patients and control; and third, if systematic differences in results could be found and traced to the type recombinant antigens used in the two assay formats.

## Results

### Antibody levels

**Figure 1:**
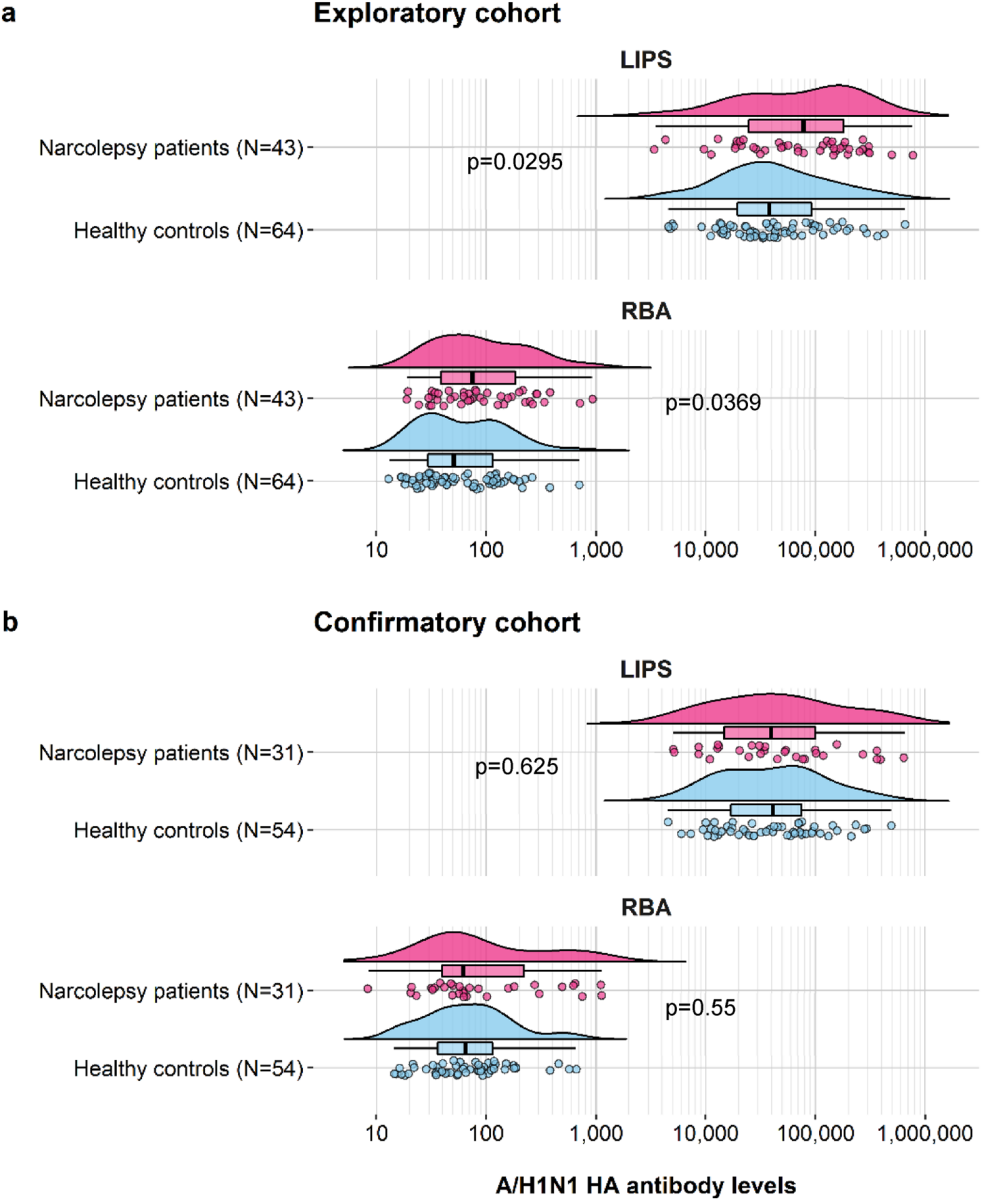
Raincloud plots of A/H1N1 HA antibody levels in subjects from the exploratory and the confirmatory study cohorts, determined using LIPS and RBA. Antibody levels in each subject are shown as circles with their overall probability density estimates in narcolepsy patients (magenta fill) or healthy controls (light blue fill). Boxplots represent the interquartile range (IQR) and median A/H1N1 HA antibody levels (solid black lines) measured in LIPS and in RBA. Panels show results from the exploratory (**a**) and the confirmatory (**b**) study cohorts. P-values are indicated between the compared groups.

A/H1N1 HA antibody levels were determined in patients and controls from both the exploratory and confirmatory study cohorts. Comparisons were performed separately for LIPS and RBA and antibody titres were found to be non-normally distributed. A/H1N1 HA antibodies were similar in range but with modestly increased median levels among narcolepsy patients compared to control subjects in the exploratory cohort both in LIPS (median arbitrary units (AU): 77,874, IQR: 24,834–182,371 vs. 37,942, IQR: 19,560–92,347, p = 0.0295) and in RBA ( median U/ml: 75, IQR: 39–185 vs. 50, IQR: 29–114, p = 0.0369) (Fig. 1a). However, levels were comparable between NT1 patients and family member controls from the confirmatory cohort both in LIPS (median AU: 44,965, IQR: 16,727–109,043 vs. 41,219, IQR: 17,123–80,778, p = 0.55) and in RBA (median U/ml: 62, IQR: 40–227 vs. 64, IQR: 36–115, p = 0.625) (Fig. 1b).

### Age

A/H1N1 HA antibody levels measured by LIPS were investigated in relation to age. In the exploratory cohort, we observed a modest inverse correlation of HA antibody levels with age in both cases and controls (R^2^ = 0.092 and 0.16, respectively). This finding was replicated in the confirmatory cohort ((R^2^ = 0.24 and 0.16, respectively). Our prior findings^33^ suggested that differences in antibody levels were most prominent in children aged less than 13 years. Accordingly, we stratified the study subjects into two age groups: children (< 13 years) and adolescents/adults (≥ 13 years). Upon stratification, we observed that HA antibody levels measured by LIPS in healthy controls were higher in younger vs older subjects in both study cohorts (Mann–Whitney p = 0.00386 and p = 0.0037) (Table 1). HA antibody levels were higher in younger vs older NT1 patients but this difference reached statistical significance only in the confirmatory cohort (p = 0.020). Compared to controls belonging to the same age group, HA antibody levels in NT1 patients were higher only in the exploratory cohort (Mann–Whitney p = 0.0419 not corrected).

**Table 1:** A/H1N1 HA antibody levels in relation to age.

Assay	Age	NT1 patients AU or U/ml median (range)	Healthy controls AU or U/ml median (range)	p1	p2	p3
**Exploratory study**						
LIPS	0–13	119,108 (21,108–763,685)	75,000 (12,501–418,785)	0.289	0.45	**0.0039**
LIPS	> 13	69,242 (3460–497,302)	27,764 (4566–654,324)	**0.0419**		
RBA	0–13	136 (24–921)	69 (18–258)	0.0882	0.138	0.426
RBA	> 13	70 (19–723)	46 (13–708)	0.12		
**Confirmatory study**						
LIPS	0–13	76,812 (25,069–3,390,545)	102,923 (27,941–1,594,069)	0.918	**0.0202**	**0.0037**
LIPS	> 13	31,055 (5109–366,663)	31,473 (4528–233,670)	0.802		
RBA	0–13	62 (21–1136)	76 (17–651)	0.605	0.761	0.835
RBA	> 13	62 (8–1116)	64 (15–463)	0.736		

LIPS and RBA results are reported according to age groups: childhood patients (exploratory study, n = 11; confirmatory study, n = 11), childhood controls (exploratory study, n = 21; confirmatory study, n = 10), adult patients (exploratory study n = 32; confirmatory study, n = 20), adult controls (exploratory study, n = 43; confirmatory study, n = 44). AU and U/ml are referred to LIPS and RBA results, respectively. Indicated p-values refer to Mann–Whitney U tests comparing A/H1N1 HA antibodies levels between: patients and age-matched controls (p1), narcolepsy childhood and adolescent/adult patients (p2), healthy childhood and adolescent/adult controls (p3).

### LIPS vs. RBA comparisons

Antibody levels were compared between LIPS and RBA. In the exploratory cohort, antibody titres correlated both in the patients (rho = 0.370, p = 0.0151) as well as the controls (rho = 0.303, p = 0.0152). In the confirmatory cohort, antibodies correlated significantly only in patients (rho = 0.664, p = 6.98e-05) but not in controls (rho = 0.203, p = 0.141) (Supplementary Figure S1).

### HLA

A/H1N1 HA antibody levels were compared between subjects carrying the HLA-DQB1*06:02:01 allele and non-HLA-DQB1*06:02:01 individuals. Increased antibody levels were related to HLA-DQB1*06:02:01 in LIPS (80,618 LU, n = 54, 37,523 LU, n = 47, p = 0.02) but not in the RBA, and only in the exploratory study (Supplementary Table S1).

### HA antibody affinity

**Figure 2:**
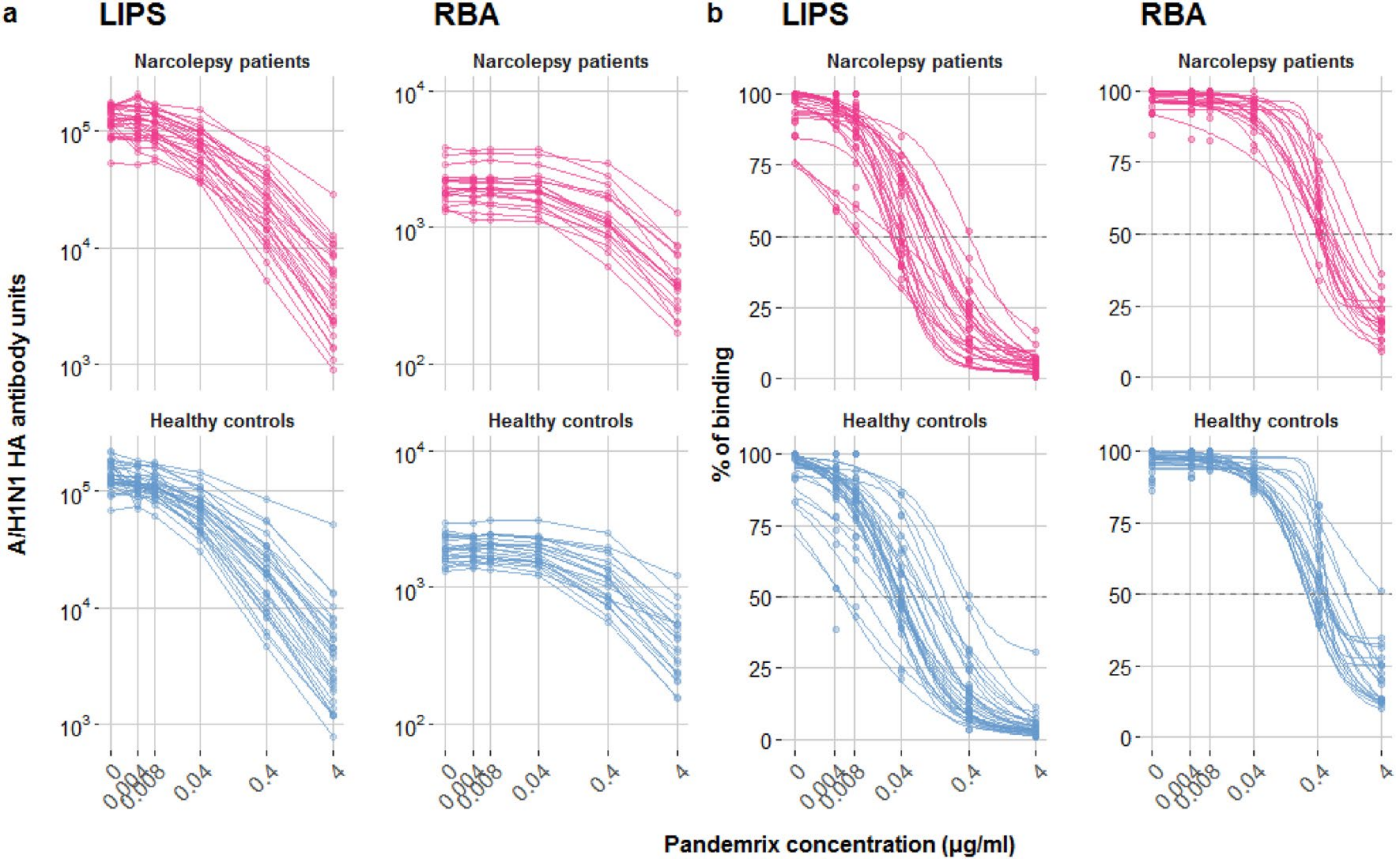
Displacement analysis of A/H1N1 HA antibodies binding in a subset of subjects from the exploratory cohort. Antibody binding was measured in the presence of increasing concentrations of Pandemrix vaccine both in LIPS (patients n = 28, controls n = 32) and in RBA (patients n = 19, controls n = 22). (**a**) Non-normalized displacement curves, showing HA antibody raw measurements (AU and cpm) with increasing competition. (**b**) Fitted curves, determined by a fitted four-parameter log-logistic function (R-package drc) on measured percentage of binding, indicated by coloured dots.

**Figure 3:**
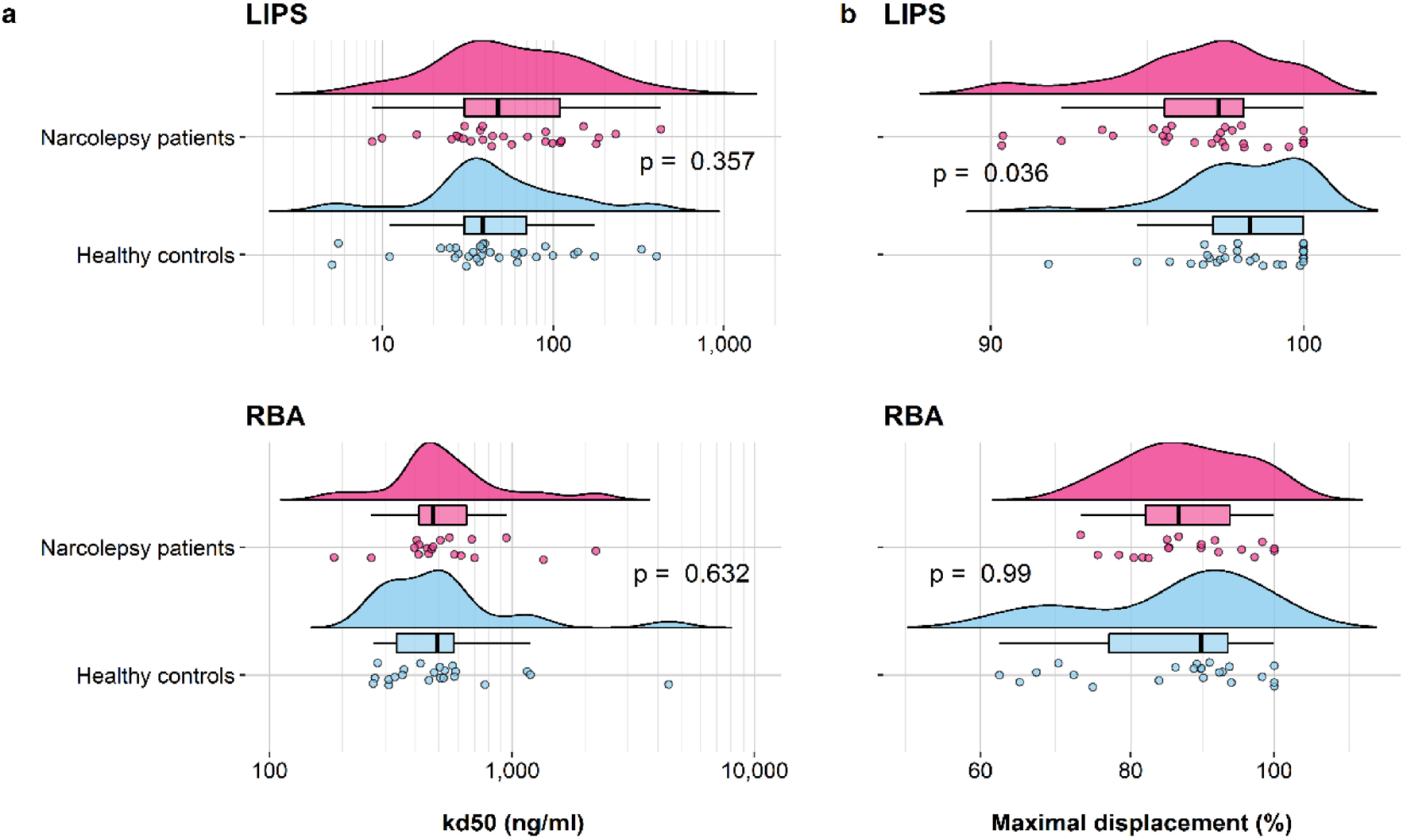
Affinity analysis of A/H1N1 HA antibodies binding in a subset of subjects from the exploratory cohort. Kd50 values were calculated as the concentration of Pandemrix vaccine necessary to displace 50% of maximum binding, while the maximal displacement values were computed as (100—the percentage of minimal binding), in the subsets of subjects described in Fig. 2 legend. Raincloud plots indicate the kd50 (**a**) or the maximal displacement (**b**) values of single samples (dots) with their overall probability density estimates and boxplots, representing the median (solid black line) and the IQR in narcolepsy patients (magenta) and healthy controls (light blue). P-values are indicated between the compared groups. Note the different x-axis scales.

The affinity of HA antibodies was evaluated in LIPS and RBA in a selection of sera from the exploratory cohort. Kd50 was determined by displacement of antibody binding using increasing concentrations of Pandemrix vaccine (Fig. 2). Since HA antibody titres were often near or at the saturation limit of our assays, all selected samples were diluted to achieve comparable levels of binding within the linear range of the assays. Kd50 were comparable between patients and controls in the LIPS (NT1: 48 ng/ml vs. control: 39 ng/ml, p = 0.357) and the RBA (NT1: 472 ng/ml vs. control: 491 ng/ml, p = 0.632) (Table 2a, Fig. 3a). Maximal displacements were higher for controls in the LIPS but comparable between the groups in the RBA (LIPS: NT1 97.2% vs. control 98.0%, p = 0.036, RBA: NT1 86.6% vs. control 89.7%, p = 0.99) (Table 2b, Fig. 3b).

**Table 2:** Affinity analysis of A/H1N1 HA antibodies.

Assay	NT1 patients ng/ml median (range)	Healthy controls ng/ml median (range)	p
a) Calculated Kd50			
LIPS	48 (9–427)	39 (8–402)	0.357
RBA	472 (184–2216)	491 (268–4429)	0.632

Assay	NT1 patients% Median (range)	Healthy controls% Median (range)	p
b) Maximal displacement			
LIPS	97.2 (90.3–100)	98.0 (70.1–100)	**0.036**
RBA	86.6 (73.2–100)	89.7 (62.5–100)	0.99

Calculated Kd50 values (**a**) and maximal displacement (**b**) of A/H1N1 HA antibodies in a subset of subjects from the exploratory cohort described in Fig. 2, using the Pandemrix vaccine as competitor both in LIPS and RBA. Indicated p-values refer to Mann–Whitney U tests comparing affinity parameters between patients and controls.

## Discussion

The report of increased narcolepsy cases following a nationwide vaccination programs with the Pandemrix flu vaccine in Sweden and Finland emerged in August 2010, with the full scope of its association with influenza vaccination surfacing in 2011–2012. The vaccine had never received approval by the FDA for distribution in the USA and the concern for the safety of its administration led to the effective discontinuation of all vaccination programs using Pandemrix in Europe and other countries after 2012 and eventually to the expiry of its marketing license in 2015.

The mechanistic association between narcolepsy and Pandemrix vaccination has proven contentious. In light of the predominant hypothesis that narcolepsy has an autoimmune origin, the specific formulation of inactivated viral components and adjuvant in the Pandemrix vaccine has been suspected of triggering the disease in genetically predisposed young individuals^34^ via the induction of autoimmunity to self-proteins via molecular mimicry. However, some of the evidence previously published regarding this point has not withstood scrutiny^35^, leaving essentially unresolved the link between Pandemrix vaccination and narcolepsy development.

To contribute towards a better understanding of the immune response in patients that developed narcolepsy after a Pandemrix vaccination, in this and previous studies we explored whether the antibody response to the flu antigens included in the vaccine formulation was quantitatively or qualitatively different from controls.

In this work in patients that developed the disease soon after the 2009 nationwide program in Sweden, we not only expand the number investigated of these rare subjects but also addressed the concern that methodological differences might impact the evaluation of flu antibodies. In particular, we explored how the characteristics of recombinant antigen used in two different immunoassays affected the measurement of antibodies to HA, a protein that in its native state has trimeric quaternary structure and is subjected to important glycosylation as a post-translational modification.

Using a novel LIPS immunoassay based on a recombinant trimeric HA antigen expressed in eukaryotic cells, in the original exploratory cohort we were able to replicate our previous observation of a broadly similar range of HA antibodies with modestly increased median levels in narcoleptic patients compared to control. In addition, HA antibodies measured by LIPS were higher in subjects carrying the HLA-DQB1*06:02:01 allele irrespective of disease status. In the second confirmatory cohort instead, we found no statistically significant difference neither in range, distribution and median HA antibody levels in NT1 patients compared to family member controls using either LIPS or RBA assays. This observation suggests that, if present, the original modest increase in median HA antibody levels might have been limited to a relatively brief temporal window after vaccination. In addition, the enrichment of HLA-DQB1*06:02:01 alleles in the family members vs the general population (72 vs 28%, respectively) might have affected the comparison HA antibodies between case and control. Overall, while observing a range of HA antibodies broadly similar in their distribution using both immunoassays, the partial correlation of binding between LIPS and RBA was suggestive of the likely detection of partially different epitopes by the two systems.

It is known that HA has a complex expression profile, with different versions of the protein present during an infection cycle, as well as major differences in glycosylation patterns that affect immune responses. The HA 3D-structure on viral particles guides antibody responses to some epitopes while hinder the exposure of others, for example in the HA stem-regions. Most studies and diagnostic procedures often prioritize simplicity and inexpensiveness of antibody assays over a thorough assessment of how immunoglobulins (vaccine- or infection induced) that bind recombinant proteins recapitulate the humoral response recognizing native antigens. In fact, the choice and type of recombinant protein adopted in immunoassay can have a major impact results when measuring antibodies including those associated with virus induced responses in a disease^36^ or vaccination setting.

In our study, while the impact of the addition of a nanoluciferase reporter on the protein structure was not assessed, both the adopted expression system and the addition of a trimerization foldon domain to the chimeric antigen were previously shown to lead to a post-translationally modified, trimeric, structurally stable recombinant HA protein that was similar to its native counterpart. Moreover, in addition to an improved antigen protein-folding, the intrinsic high luciferase activity of the NanoLuc reporter was likely associated with the observed increased sensitivity of the HA antibody LIPS compared to RBA.

Using both immunoassays in experiment of binding displacement using Pandemrix vaccine as a competitor, we determined that HA antibodies showed a comparable affinity in NT1 patient and control sera. This was in contrast to our previous study in which we observed a lower affinity of NP antibodies in NT1 patients using RBA and non-PTM NP radiolabeled antigen. Importantly, in the previous study commercially available NP proteins were used to displace antibodies instead of the Pandemrix vaccine itself^20^ as in the current study. Since Pandemrix comprises polymorphic and structurally altered NPs^21^, vaccination might have driven the emergence of antibodies that would be more difficult to displace using commercial NP antigens produced in alternative systems.

Among the limitations of our study we can count: first, the lack of information regarding the HA antibody status prior to the Pandemrix vaccination in both study cohorts, and second, the fact that patients in the confirmatory cohort were sampled in 2015–2017 i.e. a few years later compared to the exploratory cohort sampled in 2012. In the absence of additional NT1 samples collected soon after the Pandemrix vaccination, we resorted to use these samples to analyse a larger number of these very rare patients. The difference in timing of collection might have impacted on the correlation between HA antibodies and disease. In particular, the possible exposure to the pandemic flu virus in the intervening years after the vaccination might have constituted a potential confounder of immunological results. It cannot be excluded that the characteristics of dominating flu strains circulating in the years following the vaccination, including their antigenicity and sequence divergence, might have been shifted in between the sample collection of the exploratory and confirmatory cohorts. For this reason and in the absence of sequential samples from the same subjects, as mitigatory measure, we enrolled in the confirmatory cohort patients’ family members as control, reasoning that any potential exposure to a flu virus infection in NT1 patients would have been at least more likely to have affected control samples from the same family and to be synchronous. Re-immunization due to additional vaccination in both patients and family members was instead deemed unlikely, also because of the absence of state sponsored flu vaccination programs in Sweden before or after the 2009 pandemic except for the elderly.

Nevertheless, the comparable results obtained using two alternative immunoassays, of which one is ideally suited to the measurement of antibodies to conformational epitopes, make us we believe that our study main outcomes are valid: (1) quantitative differences of HA antibodies in NT1 patients compared to control were very modest and observed only in samples obtained relatively close to the vaccination (2) the affinity of HA antibodies did not differentiate NT1 patients from control.

In conclusion, while our results suggest that the HA antigen is unlikely to play a role in the association between Pandemrix vaccination and increased incidence of NT1, the mechanistic explanation of this relationship remains elusive.

## Methods

### Study populations

The current project included two narcolepsy cohorts: an exploratory and a confirmatory study group. All patients developed disease following the 2009-autumn Pandemrix vaccination and before the end of sample collection in 2012. The exploratory cohort consisted of 43 Pandemrix vaccinated NT1 patients and 64 matched population controls collected in 2012, as previously described^20,33^. The median age of patients in October of 2009 (start of vaccination) was 17 years (range 6–69 years, inter-quartile-range 12–27 years). Compared to our previous study, that included 47 patients and 80 controls, we excluded 4 patients and 16 controls who were not vaccinated with Pandemrix. The confirmatory cohort consisted of 31 Pandemrix vaccinated NT1 patients and 54 first-degree relatives as control, collected in 2015–2017 in collaboration with the Swedish Narcolepsy Association as previously described^18,37^. Patients were asked by questionnaire for year and month of acquired disease. Based on 22 answers, the median age was 15.5 years (range 6–70 years, inter-quartile-range11,75–19.5 years). No patients or controls were shared between the study-cohorts. In contrast to our previous study, that included 31 patients and 66 first-degree relatives, we now excluded 12 controls who were not Pandemrix vaccinated. The study was approved by the Regional Ethical Review Boards in Lund (Sweden) and Stockholm (Sweden), and conducted in accordance with the current guidelines. The informed consent was obtained from all participating subjects and/or their legal guardians.

### Determination of antibody affinity

To quantify the affinity of HA antibodies we used serum samples from the exploratory cohort, of these 60 were analysed conducting binding displacement experiments in LIPS and 41 in RBA. The discrepancy in the tested subjects is explained by the lower antibody levels measured by RBA that precluded the conduction of displacement experiments since at the lower displacement concentration the signal would be comparable to background noise. One control-sample was excluded from calculations in the RBA as displacement did not reach 50% as required for determination of Kd50.

### LIPS

The influenza A virus (A/California/04/2009/(H1N1)) hemagglutinin HA1 coding sequence corresponding to amino acid 1–344 was subcloned into a modified pCMVTnT-vector upstream of and in frame with a modified NanoLuc luciferase reporter (Promega, Madison WI, USA) tagged at the COOH terminus with a T4 foldon trimerization domain^38^. A recombinant trimeric luciferase-tagged HA1 recombinant antigen was then expressed by transient transfection of the plasmid into Expi293F cells (Expi293 Expression System, Thermo Fisher Scientific Life Technologies, Carlsbad, CA, USA) followed by harvest after 48 h with Passive Lysis Buffer (Promega). The recombinant trimeric luciferase-tagged HA1 was then kept frozen at − 80 °C as single-use aliquots.

For the LIPS assay, the thawed antigen was diluted in 20 mM Tris Buffer, 150 mM NaCl, 0.5% Tween-20, pH 7.4 (TBST) buffer, filtered with a Durapore PVDF 0.45-μm Millex-HV syringe filter (Millipore, Billerica, MA, USA) and adjusted for its luciferase activity to a final concentration of 4 × 10^6^ Light Units (LU)/5 μl. 1 μl replicates of each serum were incubated with 5 μl of antigen preparation into 96-deep-well plates (Beckman Coulter Inc., Brea, CA, USA) for 2 h at RT.

Immunocomplexes were captured with 2.5 μl of blocked^39^ rProtein A (GE Healthcare Europe GmbH, Freiburg, Germany) for 1 h at 4 °C with shaking. Plates were washed 5 times by dispensing 750 μl/well of TBST, centrifugation at 500 g for 3′ at 4 °C, and removal of supernatant using a micro-plate plate washer (BioTek Instruments Inc., Winooski, VT, USA). Resin pellets were transferred to an OptiPlate 96-well plate (PerkinElmer, Waltham, MA, USA) and the luciferase activity was measured after addition of 40 μl/well of Nano-Glo substrate (Promega), followed by a 2 s/well readout in a Berthold Centro XS3 Luminometer (Berthold Technologies GmbH & Co. KG, Bad Wildbad, Germany). Raw data were converted to Arbitrary Units (AU) using a positive serum as index. Sera that bound recombinant H1N1 above the linear range of the assay were serial diluted and re-tested until binding fell into the linear range, and calculated AU were corrected by multiplying for the corresponding dilution factor.

Antibody binding displacement experiments were performed by adding to test reactions Pandemrix vaccine in increasing amounts, corresponding to final concentrations of 0, 0.004, 0.008, 0.04, 0.4 and 4 μg/ml.

### In vitro transcription translation and RBA

The influenza A virus (A/California/04/2009/(H1N1)) HA gene (GeneBank accession: FJ966082) was subcloned into the pTnT-vector to enable in vitro transcription translation for expression of [^35^S]-methionine radiolabelled antigen as previously described^33,40^. The TnT SP6-coupled reticulocyte lysate system was used for protein-synthesis, and the protocol included 90 min incubation at 30 °C followed by size-restricted selection of protein-product using Nap-5 columns.

In brief, the RBA included analyses of 2.5 μl serum in duplicate. Radiolabelled proteins were diluted in assay buffer (Tris-buffered saline (pH 7.4), 0.15% (v/v) Tween 20, 0.1% (w/v) bovine serum albumin) to 400 counts per minute (cpm)/μl, and a total of 60 μl were incubated overnight with each serum sample. Immunoprecipitation was performed through addition of 50 µl protein A Sepharose to 50 μl antigen-sera sample on filter plates. Incubation, 60 min, were followed by 3 washes using Tris-buffered saline ((pH 7.4), 0.15% (v/v) Tween 20). Analyses were performed in Wallac Microbeta Trilux beta counter following addition of 50 μl Super-mix scintillation cocktail. Antibody levels were presented as in-house arbitrary Units/ml (U/ml).

Displacement experiments were performed using RBA as described above but with additional analyses included with increasing concentration of Pandemrix vaccine (0, 0.004, 0.008, 0.04, 0.4 and 4 μg/ml). The vaccine was diluted to twice the desired concentration in assay buffer, and then mixed 50/50 with radiolabelled proteins (800 cpm/μl) to obtain final concentration.

### HLA genotyping

HLA-DQB1*06:02 association in the exploratory study were determined using TaqMan assay with SNP rs9271366 (GG DQB1*06:02/, DQB1*06:02, AG DQB1*06:02/ nonDQB1*06:02 and AA nonDQB1*06:02/ nonDQB1*06:02).

HLA-DQB1*06:02 association in the confirmatory study were determined using HLA high resolution sequencing with Illumina MiSeq as previously described^18^.

### Statistical analysis

All statistical analyses were performed using the R software^41^ (https://www.R-project.org). Comparisons of median antibody titres across groups were performed using the Mann Whitney U test. For age stratification, ages in the confirmatory cohort were re-calculated back to October 2009, i.e. the initiation of Pandemrix vaccination in Sweden. The correlation between LIPS and RBA antibody titres was assessed using the Spearman’s coefficient of rank correlation test (ρ (rho)). Affinity was calculated through the determination of the half-maximal binding Kd50 (ng/ml). A four-parameter log-logistic function was constructed with y = D + (A − D)/(1 − (x/C)^D) as previously described using the drc package^20,42^ (https://CRAN.R-project.org/package=drc). The significance threshold (a) was set to 0.05 for all tests.

## Supplementary Information

Supplementary Information

## Data Availability

The datasets analysed in the current study are not publicly available due to privacy and ethical reasons but are available from the corresponding author on reasonable request.

## References

[1] Persson I (2014). Risks of neurological and immune-related diseases, including narcolepsy, after vaccination with Pandemrix: A population- and registry-based cohort study with over 2 years of follow-up. J. Intern. Med..

[2] Szakacs A, Darin N, Hallbook T (2013). Increased childhood incidence of narcolepsy in western Sweden after H1N1 influenza vaccination. Neurology.

[3] Heier MS (2013). Incidence of narcolepsy in Norwegian children and adolescents after vaccination against H1N1 influenza A. Sleep Med..

[4] Trogstad L (2017). Narcolepsy and hypersomnia in Norwegian children and young adults following the influenza A(H1N1) 2009 pandemic. Vaccine.

[5] Granath F, Gedeborg R, Smedje H, Feltelius N (2019). Change in risk for narcolepsy over time and impact of definition of onset date following vaccination with AS03 adjuvanted pandemic A/H1N1 influenza vaccine (Pandemrix) during the 2009 H1N1 influenza pandemic. Pharmacoepidemiol. Drug Saf..

[6] Feltelius N (2015). A coordinated cross-disciplinary research initiative to address an increased incidence of narcolepsy following the 2009–2010 Pandemrix vaccination programme in Sweden. J. Intern. Med..

[7] Partinen M (2012). Increased incidence and clinical picture of childhood narcolepsy following the 2009 H1N1 Pandemic vaccination campaign in Finland. PLoS ONE.

[8] Nohynek H (2012). AS03 adjuvanted AH1N1 vaccine associated with an abrupt increase in the incidence of childhood narcolepsy in Finland. PLoS ONE.

[9] Sarkanen TO, Alakuijala APE, Dauvilliers YA, Partinen MM (2018). Incidence of narcolepsy after H1N1 influenza and vaccinations: Systematic review and meta-analysis. Sleep Med. Rev..

[10] Han F (2011). Narcolepsy onset is seasonal and increased following the 2009 H1N1 pandemic in China. Ann. Neurol..

[11] Han F, Lin L, Li J, Dong XS, Mignot E (2013). Decreased incidence of childhood narcolepsy 2 years after the 2009 H1N1 winter flu pandemic. Ann. Neurol..

[12] Huang W-T (2020). Narcolepsy and 2009 H1N1 pandemic vaccination in Taiwan. Sleep Med..

[13] Wu H (2014). Symptoms and occurrences of narcolepsy: a retrospective study of 162 patients during a 10-year period in Eastern China. Sleep Med..

[14] Thannickal TC (2000). Reduced number of hypocretin neurons in human narcolepsy. Neuron.

[15] Scammell TE (2015). Narcolepsy. N. Engl. J. Med..

[16] Kornum BR (2017). Narcolepsy. Nat. Rev. Dis. Primers.

[17] Tafti M (2014). DQB1 locus alone explains most of the risk and protection in narcolepsy with cataplexy in Europe. Sleep.

[18] Lind A (2019). HLA high-resolution typing by next-generation sequencing in Pandemrix-induced narcolepsy. PLoS ONE.

[19] Melén K (2013). No serological evidence of influenza A H1N1pdm09 virus infection as a contributing factor in childhood narcolepsy after pandemrix vaccination campaign in Finland. PLoS ONE.

[20] Lind A (2017). Antibody affinity against 2009 A/H1N1 influenza and pandemrix vaccine nucleoproteins differs between childhood narcolepsy patients and controls. Viral Immunol..

[21] Vaarala O (2014). Antigenic differences between AS03 adjuvanted influenza A (H1N1) pandemic vaccines: Implications for pandemrix-associated narcolepsy risk. PLoS ONE.

[22] Ahmed SS (2015). Antibodies to influenza nucleoprotein cross-react with human hypocretin receptor 2. Sci. Transl. Med..

[23] Ramberger M (2017). CD4+ T-cell reactivity to orexin/hypocretin in patients with narcolepsy type 1. Sleep.

[24] Luo G (2018). Autoimmunity to hypocretin and molecular mimicry to flu in type 1 narcolepsy. Proc. Natl. Acad. Sci. U.S.A..

[25] Latorre D (2018). T cells in patients with narcolepsy target self-antigens of hypocretin neurons. Nature.

[26] Kornum BR (2017). Absence of autoreactive CD4+ T-cells targeting HLA-DQA1*01:02/DQB1*06:02 restricted hypocretin/orexin epitopes in narcolepsy type 1 when detected by EliSpot. J. Neuroimmunol..

[27] Jiang W (2019). In vivo clonal expansion and phenotypes of hypocretin-specific CD4 + T cells in narcolepsy patients and controls. Nat. Commun..

[28] Cogswell AC (2019). Children with Narcolepsy type 1 have increased T-cell responses to orexins. Ann. Clin. Transl. Neurol..

[29] Schinkelshoek MS (2019). H1N1 hemagglutinin-specific HLA-DQ6-restricted CD4+ T cells can be readily detected in narcolepsy type 1 patients and healthy controls. J. Neuroimmunol..

[30] Liu E, Eisenbarth GS (2007). Accepting clocks that tell time poorly: Fluid-phase versus standard ELISA autoantibody assays. Clin. Immunol..

[31] Pihoker C, Gilliam LK, Hampe CS, Lernmark A (2005). Autoantibodies in diabetes. Diabetes.

[32] Winqvist O, Karlsson FA, Kämpe O (1992). 21-hydroxylase, a major autoantigen in idiopathic Addison’s disease. Lancet.

[33] Lind A (2014). A/H1N1 antibodies and TRIB2 autoantibodies in narcolepsy patients diagnosed in conjunction with the Pandemrix vaccination campaign in Sweden 2009–2010. J. Autoimmun..

[34] Bomfim IL (2017). The immunogenetics of narcolepsy associated with A(H1N1)pdm09 vaccination (Pandemrix) supports a potent gene–environment interaction. Genes Immun..

[35] De la Herrán-Arita AK (2014). Retraction of the research article: ‘CD4^+^ T cell autoimmunity to hypocretin/orexin and cross-reactivity to a 2009 H1N1 influenza A epitope in narcolepsy’. Sci. Transl. Med..

[36] Secchi M (2020). COVID-19 survival associates with the immunoglobulin response to the SARS-CoV-2 spike receptor binding domain. J. Clin. Investig..

[37] Wallenius M (2019). Autoantibodies in Pandemrix®-induced narcolepsy: Nine candidate autoantigens fail the conformational autoantibody test. Autoimmunity.

[38] Lu Y, Welsh JP, Swartz JR (2014). Production and stabilization of the trimeric influenza hemagglutinin stem domain for potentially broadly protective influenza vaccines. Proc. Natl. Acad. Sci. U.S.A..

[39] Williams AJK, Norcross AJ, Chandler KA, Bingley PJ (2006). Non-specific binding to protein A Sepharose and protein G Sepharose in insulin autoantibody assays may be reduced by pre-treatment with glycine or ethanolamine. J. Immunol. Methods.

[40] Grubin CE (1994). A novel radioligand binding assay to determine diagnostic accuracy of isoform-specific glutamic acid decarboxylase antibodies in childhood IDDM. Diabetologia.

[41] R Core Team. *R: A Language and Environment for Statistical Computing*. (R Foundation for Statistical Computing, 2020).

[42] Ritz C, Baty F, Streibig JC, Gerhard D (2015). Dose-response analysis using R. PLoS ONE.

