# Supplementary material for: A/H1N1 hemagglutinin antibodies show comparable affinity in vaccine-related Narcolepsy type 1 and control and are unlikely to contribute to pathogenesis
Source: Sci Rep. 2021 Feb 18;11:4063. doi: 10.1038/s41598-021-83543-z (PMC7893011; doi:10.1038/s41598-021-83543-z)
Supplement: Supplementary file 1 — Supplementary Information [file 41598_2021_83543_MOESM1_ESM.pdf]

A/H1N1 hemagglutinin antibodies show comparable affinity in vaccine-related Narcolepsy type 1 and control and are unlikely to contribute to pathogenesis

Alexander Lind[1§], Ilaria Marzinotto[2§], Cristina Brigatti[2], Anita Ramelius[1], Lorenzo Piemonti[2], Vito Lampasona[2*]

[1] Department of Clinical Sciences, Lund University/Clinical Research Center (CRC), Skåne University Hospital SUS, Malmö, Sweden
[2] San Raffaele Diabetes Research Institute, IRCCS Ospedale San Raffaele, Via Olgettina 60, 20132 Milan, Italy

Supplementary Materials

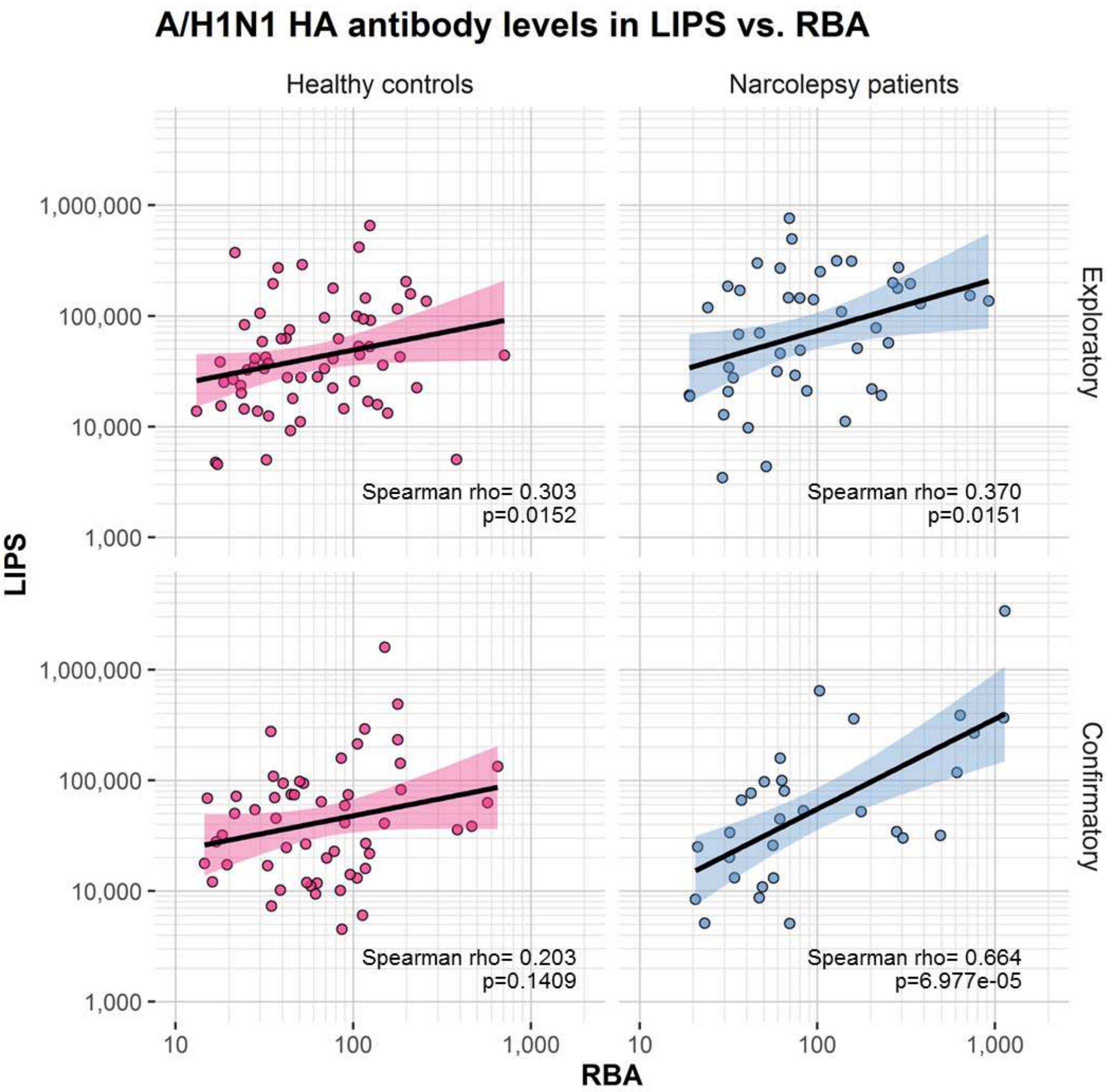

Supplementary Figure S1. Scatterplot of A/H1N1 HA antibody titres measured by LIPS vs. RBA
Antibody levels were compared in each subgroup, healthy controls (magenta) and narcolepsy patients (light blue) from both the exploratory and the confirmatory cohort. Each measurement is shown as a circle, the solid black line indicates the regression line and the coloured area its 95% confidence interval. The Spearman's coefficient rho and the p-value of each comparison are reported on the relative panel.

Supplementary Table S1. H1N1 HA antibodies in relation to HLA-DQB1*06:02:01

A/H1N1 HA antibodies levels according to the association and non-association with HLA-DQB1*06:02:01. Comparisons of titres were performed among subjects from both the exploratory (HLA associated: n=54 and non-associated: n=47) and the confirmatory cohort (HLA associated: n=69 and non-associated: n=16), regardless of patient or control status. LU and U/ml are referred to LIPS and RBA results, respectively.

Exploratory Study

Assay	HLA associated	HLA Non-associated	p
	median (range)	median (range)	
LIPS	80618 (3460 - 763685)	37523 (4566 - 291903)	0.02
RBA	69 (19 - 921)	52 (13 - 381)	0.27

Confirmatory Study

Assay	HLA associated	HLA Non-associated	p
	median (range)	median (range)	
LIPS	45543 (4528 - 3390545)	24948 (6066 -1594069)	0.24
RBA	62 (15 – 1136)	72 (8 - 386)	0.91
